# Attenuation of Hyperlipidemia by Medicinal Formulations of *Emblica officinalis* Synergized with Nanotechnological Approaches

**DOI:** 10.3390/bioengineering10010064

**Published:** 2023-01-04

**Authors:** Puttasiddaiah Rachitha, Krupashree Krishnaswamy, Renal Antoinette Lazar, Vijai Kumar Gupta, Baskaran Stephen Inbaraj, Vinay Basavegowda Raghavendra, Minaxi Sharma, Kandi Sridhar

**Affiliations:** 1 P.G. Department of Biotechnology, Teresian College, Siddarthanagar, Mysuru 570011, India; 2 Biochemistry Department, Central Food Technological Research Institute, Mysore 570006, India; 3 Biorefining and Advanced Materials Research Center, Scotland’s Rural College (SRUC), Edinburgh EH9 3JG, UK; 4 Department of Food Science, Fu Jen Catholic University, New Taipei City 242 05, Taiwan; 5 Haute Ecole Provinciale de Hainaut-Condorcet, 7800 Ath, Belgium; 6 Department of Food Technology, Koneru Lakshmaiah Education Foundation Deemed to be University, Vaddeswaram 522502, India

**Keywords:** *Emblica officinalis*, phytochemicals, hyperlipidemia, pharmacology, nanoformulation

## Abstract

The ayurvedic herb *Emblica officinalis* (*E. officinalis*) is a gift to mankind to acquire a healthy lifestyle. It has great therapeutic and nutritional importance. *Emblica officinalis*, also known as Indian gooseberry or Amla, is a member of the Euphorbiaceae family. Amla is beneficial for treating illnesses in all its forms. The most crucial component is a fruit, which is also the most common. It is used frequently in Indian medicine as a restorative, diuretic, liver tonic, refrigerant, stomachic, laxative, antipyretic, hair tonic, ulcer preventive, and for the common cold and fever. Hyperlipidemia is also known as high cholesterol or an increase in one or more lipid-containing blood proteins. Various phytocompounds, including polyphenols, vitamins, amino acids, fixed oils, and flavonoids, are present in the various parts of *E. officinalis*. *E. officinalis* has been linked to a variety of pharmacological effects in earlier studies, including hepatoprotective, immunomodulatory, antimicrobial, radioprotective, and hyperlipidemic effects. The amla-derived active ingredients and food products nevertheless encounter challenges such as instability and interactions with other food matrices. Considering the issue from this perspective, food component nanoencapsulation is a young and cutting-edge field for controlled and targeted delivery with a range of preventative activities. The nanoformulation of *E. officinalis* facilitates the release of active components or food ingredients, increased bioaccessibility, enhanced therapeutic activities, and digestion in the human body. Accordingly, the current review provides a summary of the phytoconstituents of *E. officinalis*, pharmacological actions detailing the plant *E. officinalis*’s traditional uses, and especially hyperlipidemic activity. Correspondingly, the article describes the uses of nanotechnology in amla therapeutics and functional ingredients.

## 1. Introduction

The most prevalent type of dyslipidemia, hyperlipidemia, is caused by increased blood lipid levels, including triglycerides and cholesterol. The early onset of atherosclerosis and its cardiovascular complications are predicted to continue to be significantly influenced by hyperlipidemia. Globally available cholesterol-lowering drugs may be expensive and have side effects, making herbal medicines an alternative in cases where cardiovascular diseases are the primary cause of death [1,2].

In the ancient Indian medical system known as Ayurveda, *Emblica officinalis* (*E. officinalis*) holds a revered place. It is the first tree ever made in the history of the universe. It belongs to the Euphorbiaceae family, according to ancient Indian mythology. commonly known as *Phyllanthus emblica*, Amla, or Indian gooseberry [3]. In Ayurveda, the fruit of the *E. officinalis* plant is frequently used to boost immunity. Cancer, diabetes, liver disease, ulcers, heart problems, anemia, and a number of other diseases can all be helped by the chemical constituents of *P. emblica*. Additionally, it helps with lowering cholesterol levels, ophthalmic disorders, and improving memory [4,5].

The hypoglycemic medications on the market all have one or more side effects. According to WHO recommendations, it can be difficult to find new cholesterol-lowering drugs made from herbal plants that have negligible or no side effects [6]. Interestingly, there is a paucity of information on the effectiveness of EO in lowering cholesterol. Nanocoatings made from Amla essential oil appear to extend the shelf life of fruits [7]. In addition, nanoencapsulated amla provides nutraceuticals and functional foods to improve human health. The therapeutic and traditional potential of amla can be fully utilized by nanoencapsulation of the active components of amla for target delivery, enhanced bioavailability, and increased bioactivity [8].

The present review covers hyperlipidemia, different types of hyperlipidemia, biochemical profile of *E. officinalis*, and attenuation of hyperlipidemic activity of *Emblica officinalis* phytochemicals. With these most significant advances, theranostic treatments for hyperlipidemia could become very efficient in the near future.

## 2. Hyperlipidemia

A prevalent condition of lipid metabolism known as hyperlipidemia is brought on by elevated triglycerides and total cholesterol levels. According to Panahi, et al. [9], hyperlipidemia is characterized by elevated triglycerides, free fatty acid (FFA), low-density lipoprotein (LDL), cholesterol, and apolipoprotein B (apoB) levels as well as a reduced plasma concentration of high-density lipoprotein (HDL) cholesterol. This condition is the primary risk factor for cardiovascular diseases (CVDs), such as coronary heart disease (CHDs) and the normal blood cholesterol level [10].

### 2.1. Causes of Hyperlipidemia

The plasma lipids result from either a primary genetic defect, a secondary diet, medication, or disease [11]. The vast majority of cholesterol is produced internally. 3-hydroxy-3-methyl-glutaryl coenzyme A (HMG CoA) reductase is the rate-limiting enzyme for the synthesis of endogenous cholesterol, and it offers a crucial approach to pharmacologic therapy [12]. The liver packages endogenously produced cholesterol and triglycerides into soluble particles. The core of soluble particles, which are rich in triglycerides and cholesterol ester, is encased in a phospholipid membrane made up of different apolipoproteins. The characteristics of apolipoproteins include receptor recognition sites, which facilitate the specific metabolism of these particles by lipoprotein lipase in conjunction with the metabolism of triglycerides. In the beginning, the liver creates triglyceride-rich, very-low-density lipoproteins (VLDL). Low-density lipoproteins (LDL) are made from these particles, which shrink in size, or from oxidized LDL, broken down by the lipoprotein lipase enzyme [13]. This LDL can be absorbed by particular receptors, used by macrophages or the liver, and removed from circulation. High-density lipoprotein (HDL) particles, which are cholesterol-rich and have antioxidant properties, start reverse cholesterol transport [14].

### 2.2. Types of Hyperlipidemia

The American Heart Association (AHA) divides hyperlipidemia into two categories: primary hyperlipidemia, which is based on genetics, and secondary hyperlipidemia, which is brought on by conditions such as diabetes, liver, kidney, thyroid, and Cushing’s syndrome, as well as estrogen therapy, alcohol consumption, and drug use that alters lipid metabolism [15,16]. Additionally, in accordance with the “Fredrickson” classification, there have been five different types of hyperlipidemia: Type I (raised cholesterol with high triglyceride levels), Type II (high cholesterol with normal triglyceride levels), Type III (raised cholesterol and triglycerides), Type IV (raised triglycerides, atheroma, and raised uric acid), and Type V—raised triglycerides [17,18], as shown in Table 1.

The specific classes of triglyceride-rich lipoprotein particles that accumulate in plasma, such as VLDL, chylomicrons, or intermediate density lipoprotein, help to distinguish these types of hyperlipidemia (IDL). Elevated VLDL density and chylomicron are characteristics of simple HTG, or HLP type 4. Elevated VLDL and LDL concentrations identify HLP type 2. Furthermore, patients with all types of HTG typically exhibit decreased HDL cholesterol [1]. Starc [19] represented epidemiological studies indicated the risk for coronary heart disease (CHD) starts to rise at cholesterol levels above 200 mg/dL; as a result, scientists think the relationship between atherogenesis and plasma cholesterol is linear over the entire range of cholesterol concentrations. Given the existence of a CHD risk threshold, concentrations above this point are referred to as hypercholesterolemia [20].

The connection between plasma fatty substances, such as triglyceride levels, and the onset of atherosclerosis has proven challenging to prove. According to Nelson, 2013 [14], triglyceride levels and the risk of CHD are closely related. Additionally, plasma triglyceride levels are positively correlated with risk for CHD. According to research from Harchaoui et al. [21], triglyceride concentrations lose their predictive power when data are subjected to multifactorial analysis, so we considered other risk factors. Patients with hypertriglyceridemia may experience accelerated atherosclerosis for unknown reasons. Furthermore, elevated triglyceride levels may be more highly predictive of CHD when present or absent than the degree of elevation. The fact that hypertriglyceridemia is present in the majority of patients with disproportionate CHD thus summarizes the situation [21]. The reduction of hepatic cholesterol level causes a decrease in VLDL cholesterol level in the blood [22].

### 2.3. Pathophysiology

Studies on the pathophysiology of hyperlipidemia fall into two categories at their core. Hypertriglyceridemia results from a defect in lipid metabolism, and hyperchylomicronemia is brought on by a defect in lipoprotein lipase activity or the absence of the surface apoprotein CII31 [23]. According to Karr 2017 [24], postprandial absorption of chylomicrons from the gastrointestinal tract occurs 30 to 60 min after the consumption of meals high in fat and raises serum triglycerides. Low LPL activity ultimately causes hyperlipidemia by increasing the production of VLDL cholesterol. Patients with diabetes mellitus reported seeing this.

Additionally, hypercholesterolemia manifests nephrotic syndrome, a common pathway for the synthesis of cholesterol, and albumin lowers oncotic pressure, resulting in increased cholesterol synthesis [25]. Several medications, including progestins, thiazide diuretics, glucocorticoids, Î2 blockers, isotretinoin, protease inhibitors, cyclosporine, mirtazapine, and sirolimus, have been reported by Hill and Bordoni [26] to increase lipid levels as secondary forms of hyperlipidemia. The lack of LDL degradation by cells as a result of hypercholesterolemia is due to the LDL-R complex’s inability to bind LDL and the uncontrolled production of cholesterol.

## 3. Biochemical Profiling of Amla

Medicinal remedies have served as the foundation for sophisticated traditional medicine and are extremely important to humankind in terms of basic therapeutics. Natural products are a gift for improving health and are great starting points for developing new drugs. International public health is a specialty of the United Nations and is the focus of the World Health Organization [27]. As stated by the WHO, conventional medicine is the primary source of care for 80% of the world’s population, which is significant for health care. The World Health Organization (WHO) is enjoining researchers to encourage the effective use of herbal medicine in developing nations’ health programs. Currently, herbal plants are the “local heritage of global significance”, contributing more to the healthcare system than chemical drugs [28].

The Ayurvedic valuable tree *E. officinalis* or amla has long been recognized for its therapeutic and pharmacological significance. *E. officinalis* is a member of the Euphorbiaceae family; it is also known by the common names Amla and *Phyllanthus emblica* in the botanical world [29,30]. The summary of the taxonomy of common names of *E. officinalis* is presented in Table 2. Specifically, central and southern India, Sri Lanka, southern China, Pakistan, Bangladesh, the Mascarene Islands, Malaysia, and tropical Southeastern Asia are home to the *E. officinalis* species. The *E. officinalis* appears to be a large tree with a height of 8 to 18 m, but in India, *E. officinalis* trees can be found throughout tropical forests that rise up to 4500 feet on hills [31,32]. The botanical description of *E. officinalis* is examined in Table 3 [29,33], which lists various parts of the plant, including fruits, leaves, seeds, bark, and flowers, that are used for various pharmacological effects. The most studied plant is *E. officinalis*, and reports indicate that it contains a variety of chemical components, such as gallic acid, amino acids, flavone glycosides, phenolic glycosides, flavonol glycosides, sesquiterpenoids, nor sesquiterpenoids, and rich fiber, carbohydrate, iron, tannins, alkaloids, and phenolic compounds. According to Singh et al. [34], the nutritional value of *E. officinalis* fruit contains significantly more minerals, proteins, and amino acids such as glutamic acid, proline, aspartic acid, alanine, cystine, and lysine than other fruits such as apple, lime, pomegranate, and grape. It is also said to be the richest source of vitamin C compared to other fruits, as represented in Table 4 [35,36,37,38].

### 3.1. Phytochemistry

**Figure 1:**
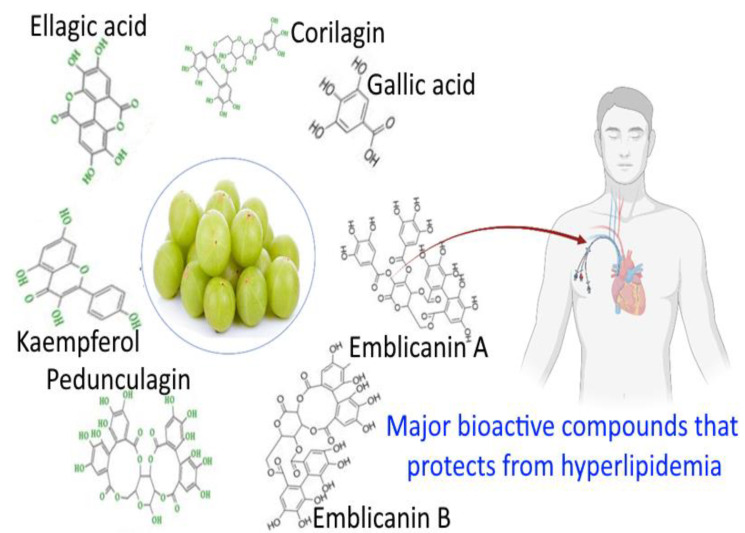
Major phytochemical constituents of *E. officinalis* that protect from hyperlipidemia.

The *E. officinalis* fruit’s most prevalent ingredient is ascorbic acid. Phosphatides, fixed oils, phosphatides, essential oils, tannins, minerals, vitamins, and amino acids are some additional phytochemicals associated with this plant. The *E. officinalis* is good source of phytochemicals (Table 5). Methyl gallate, luteolin, corilagin, isostrictiniin, gallic acid, ellagic acid, chebulinic acid, and chebulagic acid are the major polyphenolic compounds in *E. officinalis* [5,39]. Fruit pulp has been found to contain tannins such as phyllaemblicin B, emblicanin A, B, punigluconoin, and pedunculagin [40]. There have been reports of linolenic, oleic, stearic, palmitic, and myristic acids in *E. officinalis*, as well as by-products of organic acids such as citric acids and sugars such as glucose, fructose, myo-inositol, galacturonic acid, arabinosyl, rhamnosyl, xylosyl, and glucosyl. Many compounds’ isolates obtained from these plants include amlaic acid, arginine, aspartic acid, astragallin, -carotene, -sitosterol, chebulagic acid, chebulic acid, chebulinic acid, corilagic acid, corilagin, ellagic acid, emblicol, gibberellins, glutamic acid, glycine, histidine, and isoleucine [41,42,43,44,45,46] and gallic acid, 1,6-di-O-galloyl-d-glucose, 3,6-di-O-galloyl-d-glucose, corilagin, 3-ethylgallic acid (3-ethoxy-4,5-dihydroxybenzoic acid), isostrictiniin, kaempferol-3-O-L-(6’-methyl)- rhamnopyranoside [47,48,49]. In addition to isolating -rhamnopyranoside from *E. officinalis*, researchers have also isolated a number of phenolic compounds from the fruit juice of the plant, including mucic acid 2-O-gallate, L-malic acid 2-O-gallate, mucic acid 1,4-lactone 2-O-gallate, mucic acid 1,4-lactone 5-O-gallate, mucic acid 1,4-lactone 3-O-gallate, and mucic acid [50]. The phenolic glycosides 2-carboxylmethylphenol1-O-d-glucopyranoside and 2,6-dimethoxy-4-(2-hydroxyethyl) phenol 1-O-d-glucopyranoside were combined with phyllaemblicin-A, B, and C, phyllaemblic acid (methyl ester of highly oxygenated nor bisabolane), and phyllaemblic acid C and phyllaemblicin D [51,52]. Gallic acid, methyl gallate, 1,2,3,4,6-penta-O-galloylglucose, two newly discovered acrylated flavanones glycosides, (S)-eriodictyol 7-O-(6’-O-trans-p coumaroyl)—d-glucopyranoside, and luteolin-4’-O-neohesperidoside were also isolated from the leaves of *E. officinalis*. Furthermore, *E. officinalis* contains six ellagitannins and phyllanemblinins A–F [53,54]. Several new sterols, including trihydroxysitosterol and 5,6,7-acetoxysitosterol, were discovered in the branches and leaves of *E. officinalis* [55,56]. According to analytical reports, the pulp of *E. officinalis* seeds has a high phenolic content, and its seeds are primarily made up of tannins. In the pulp and seeds of *E. officinalis*, coumaric acid, myricetin, caffeic acid, gallic acid, and quercetin have all been identified [57]. Among them, gallic acid, myricetin, kaempferol, Emblicanin A and B, chebulagic acid, ellagic acid, pedunculagin, and corilagin are the major compounds that protect from hyperlipidemia, as shown in Figure 1.

### 3.2. Pharmacological Activity of Amla

**Figure 2:**
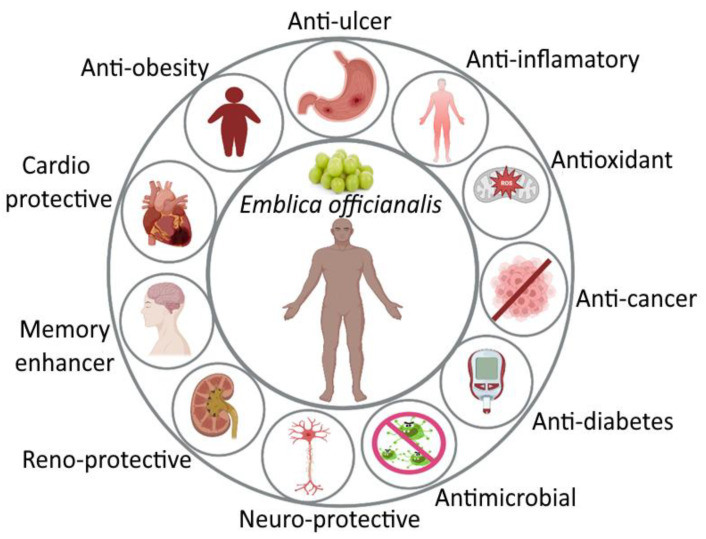
Different pharmaceutical activities of *E. officinalis*.

In light of the medicinal and pharmaceutical qualities of *E. officinalis*, every part is beneficial. According to research by Krishnaveni and Mirunalini [3], *E. officinalis* has antioxidant, antimicrobial, anti-inflammatory, anticancer, antiulcer, antidiabetic, memory enhancer, cardioprotective, neuroprotective, neuroprotective, antidiarrheal, renoprotective, and immunomodulatory potential, as shown in Figure 2, and major phytoconsituents present in *E. officinalis* and its pharmaceutical effects point toward anti-hyperlipidemia properties, as shown in Table 5 [5,58]. It also has positive effects on hyperlipidemia, osteoporosis, and a number of reactive oxygen species that can lead to oxidative stress and fundamental cell damage in the body.

Large quantities of polyphenols, tannins, and other phytochemicals found in *E. officinalis* can lessen oxidative damage to cells. The natural antioxidants of *E. officinalis* play a significant role in the activity of free radical scavengers, and methanolic seed extract and pulp extract of *E. officinalis* show promising 1,1, diphenyl-2-picryl-hydrazil (DPPH) free radical scavenging activity in a concentration-dependent manner [36,59]. There is strong considerable potential for ferric reduction, free radical scavenging, and ROS (reactive oxygen species) inhibition in the water extract of *E. officinalis* fruit [60].

Different solvent systems were used to test *E. officinalis*’ antimicrobial activity, and it was found to have antifungal properties against *Aspergillus* sps. [61]. Fruit ethanolic and acetone extract demonstrated activity against *Candida albicans* and *Fusarium equiseti*. The antibacterial activity against *Staphylococcus* was demonstrated using the zone inhibition method, and the tube dilution method significantly reduced the colony counts of *Escherichia coli*, *Staphylococcus aureus*, *Klebsiella pneumoniae*, and *Pasteurella multocida* [62,63,64]. The phytochemical in *E. officinalis* called pentagalloyl glucose has anti-influenza properties. WST-1 assay, plaque-forming unit assay, time of-addition assay, and hemagglutination inhibition (HI) assay were used to evaluate a virus replication with a dual mode of action [65].

In Sprague-Dawley rats exposed to both acute and chronic inflammation caused by carrageenan and cotton pellets, the water extract of *E. officinalis* was discovered to exhibit anti-inflammatory effects by minimizing paw volume in the case of acute inflammation and myeloperoxidase activity, granulomatous tissue mass, and plasma extravasation in the case of chronic inflammation [66]. Histopathological studies were used to investigate the hepatoprotective activity of *E. officinalis*. Liver-protective behavior HepG2 cells were used to test the efficacy of *E. officinalis* against tert-butyl hydroperoxide (t-BH)-induced toxicity, and rats were used to test the efficacy of a 50% hydroalcoholic extract of fresh *E. officinalis* fruit against chronic toxicity brought on by carbon tetrachloride and thioacetamide. Nephroprotective properties reduced the elevated levels of thiobarbituric acid-reactive substance in the serum, renal homogenate, and creatinine and urea nitrogen in aged rats [67,68]. The forced swim test (FST) and tail suspension test (TST) with Swiss albino mice were used to test the aqueous extract of fruits from *E. officinalis* for its antidepressant activity. The results revealed a significant decrease in depression. The result was that aged mice with improved memory (elevated plus maze and passive avoidance apparatus) had lower total serum cholesterol levels and higher brain cholinesterase activity [69]. In albino rats, *E. officinalis* was found to have immunomodulatory activity as evidenced by increases in hemagglutination antibody titer, macrophage migration index, hypersensitivity reaction, respiratory burst activity of the peritoneal macrophages, total leukocyte count, percentage lymphocyte distribution, serum globulin, and relative lymphoid organ weight. It is also able to stimulate humoral and cell mediated immunity as well as macrophage phagocyte [70]. On type II diabetes, triglycerides (TG), and the liver-specific enzyme alanine transaminase, the aqueous fruit extract of *E. officinalis* was assessed (ALT). According to this study, alloxan-induced diabetic rats could significantly lower their blood glucose levels when given an aqueous fruit extract dose of 200 mg/kg body weight [71]. Compared to control and extract-treated diabetic rats, oral administration of the aqueous extract (350 mg/kg body weight) significantly decreased serum glucose levels, glycosylated hemoglobin, insulin, cholesterol, triglycerides, HDL-cholesterol, protein, urea, and creatinine [72].

*E. officinalis* has the potential to lower cholesterol because it naturally contains flavonoids and other phytochemicals. Several clinical studies showed significant drops in C-reaction protein (CRP), low-density lipoprotein, and total cholesterol [73]. Variya et al. [74] assessed the hypolipidemic effect of *E. officinalis* and compared it to the standard simvastatin in patients with type-II hyperlipidemia. Treatment with *E. officinalis* resulted in significantly lower levels of total cholesterol, LDL cholesterol, and triglycerides as well as noticeably higher levels of the common medication simvastatin [75].

Polyphenols from *E. officinalis* have also been shown to protect gastrointestinal organs. Because *Helicobacter pylori* is a pathogen, one of the potential effects of amla’s bioactive compounds is the competitive inhibitor of clarithromycin-resistant strains in vitro [76]. Studies using animals reported relevant results as well. In order to induce gastrointestinal ulcers in mice, Al-Rehaily et al. [77] used a variety of techniques, including ligating the pylorus, administering indomethacin and necrotizing agents (25% NaCl, 0.2 M NaOH, and 80% ethanol), and inducing hypothermia. These techniques included studying the antisecretory and antiulcer activities of *E. officinalis* extract. Using the pylorus-ligated and necrotizing agent-intoxicated ulcer methods, both doses (250 and 500 mg/kg) decreased gastric secretion, intraluminal bleeding, ulcer index, and gastric lesions. Only the animals receiving treatment with 500 mg/kg for the indomethacin-induced ulcer method had a significantly lower ulcer index than animals in the control group (treated only with indomethacin).

### 3.3. Antihyperlipidemic Activity of Emblica officinalis

**Figure 3:**
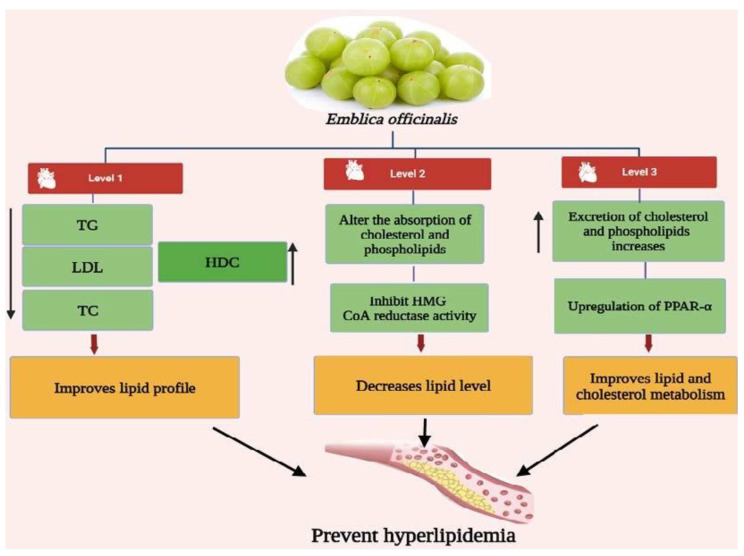
Anti-hyperlipidemia activity of amla bioactive compounds. Figure 3 is adapted from Gul et al. [80] (Copyright © 2022 by authors), which is an open access article distributed under the terms and conditions of the Creative Commons Attribution (CC BY) license. TG, triglyceride; HDL, high-density lipoprotein; LDL, low-density lipoprotein; TC, total cholesterol; PPAR-α, peroxisome proliferator-activated receptors-α.

According to reports, *E. officinalis* fruit has significant anti-hyperlipidemic, hypolipidemic, and antiatherogenic effects [78]. In patients with type II hyperlipidemia, treatment with *E. officinalis* resulted in a significantly lower level of total cholesterol (TC), low-density lipoprotein (LDL), triglyceride (TG), and very-low-density lipoprotein (VLDL), as well as a significantly higher level of high-density lipoprotein (HDL) (Figure 3). Studies conducted in vitro and in vivo using cholesterol-fed rats and Cu^2+^-induced LDL-oxidation established the anti-hyperlipidemic activity of extract from *E. officinalis* and demonstrated a significant reduction in total and free cholesterol levels in a dose-dependent manner [79]. Given that oxidized LDL is a key enzyme in atherosclerosis, administration of *E. officinalis*’ potential antioxidant property resulted in a decrease in oxidized LDL levels in cholesterol-fed subjects, as shown in Figure 3 [80].

Another study revealed elevated lecithin-cholesterol acyltransferase and hepatic 3-hydroxy 3-methylglutaryl coenzyme A (HMG-CoA) reductase inhibition as anti-hyperlipidemic effects (LCAT). Flavonoids, which prevent the synthesis and deterioration of lipids, are responsible for this effect [81]. Oral administration of *E. officinalis* extracts in a dose of 500 mg twice daily for 12 weeks significantly lowered total cholesterol, LDL cholesterol, and high-sensitive creatine kinase (hr-CRP) levels, according to a study on animals that used class one obese subjects with body weights between 250 and 350 g. *E. officinalis* showed significant antiatherosclerotic action through a decrease in serum and hepatic cholesterol content, serum triglyceride, phospholipids, and LDL cholesterol in a high-cholesterol-fed rabbit model. Platelet aggregation induced by ADP and collagen was also decreased, and diabeto-cardiac malaise was eventually reduced [73]. Growing fructose consumption in the diet has been linked to a higher risk of obesity and related metabolic syndromes in Western countries. Increased intake of fructose alters several signaling cascades, including NF-, TNF-, JNK-1, PTP-1B, PTEN, LXR, FXR, and SREBP-1c [82]. The SREBP-1 expression, total cholesterol, TG level, and metabolic issues associated with high fructose levels are all improved by the supplement’s high polyphenol content from *E. officinalis*. According to reports, *E. officinalis* inhibits the level of MDA in the liver and controls the expression of the COX-2, bax, NF-, and bcl-2 markers [83]. Previous findings have shown that *E. officinalis* therapies increase lipid metabolism and regulate the expression of proteins such as PPAR-α, which is involved in fatty acid-oxidation, FXR, and LXR involved in lipid metabolism, as well as insulin-induced gene-2 to reduce fructose-induced metabolic syndrome and prevent the maturation of steroyl CoA desaturase-1 and SREBP-1, which are involved in the synthesis of TG. Additionally, RAW 264.7 cell lines’ expression of CD36 scavenger receptor was markedly reduced to prevent foam cell formation by these mechanisms [57]. The major contributors to *E. officinali’s* antihyperlipidemic activity are its polyphenols and functional products. Gallic acid, Vitamin C, Emblicanin A and B, apigenin, ellagic acid, and 3-hydroxy-3-methylglutaryl-CoA myricitine, among other antioxidants, polyphenols, and phenolic acids, play a significant role in lowering hyperlipidemia [84,85,86,87]. As these molecules are less stable with different environmental conditions or pH and less soluble in water, they are reported to have less bioavailability or poor assimilation of major constituents. This is the main drawback of these polyphenols and functional products [87,88]. Consequently, nanoformulation strategies can increase the bioavailability of phenolic acids and functional products.

### 3.4. Nanoparticulate Carrier System for the Treatment of Hyperlipidemia

**Figure 4:**
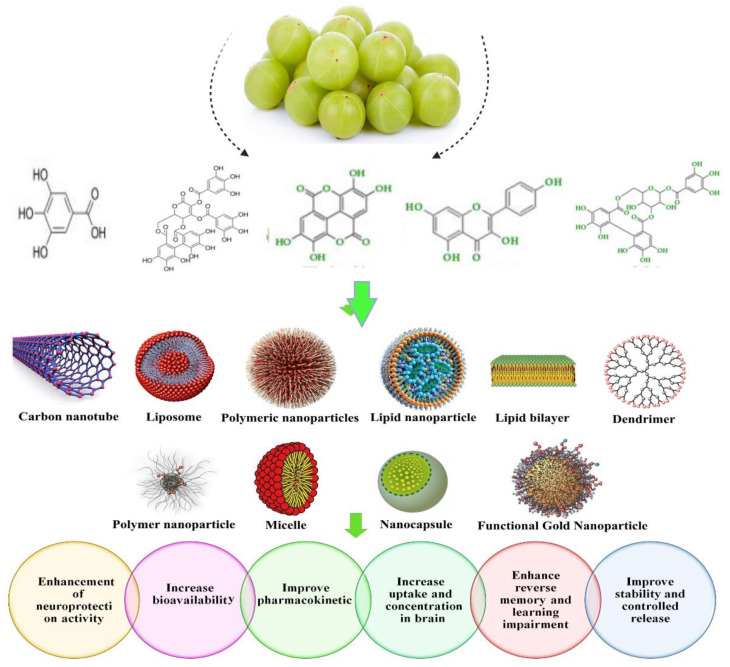
Nanoformulations from natural products and their advantages at a target delivery site. Figure 4 is adapted from Moradi et al. [91] (Copyright © 2020 by authors), which is an open access article distributed under the terms and conditions of the Creative Commons Attribution (CC BY) license.

Nanotechnology is one of the emerging technologies that influence human life in different approaches that assist in overcoming the multiple limitations of various diseases, especially hyperlipidemia. Nanoformulations have become a novel profitable approach for increasing the bioavailability of poor soluble drugs [84,89]. These nanoformulations have several unique qualities that make them more valuable for the drug delivery system. Diverse nanostructures include solid lipid nanoparticles (SLNs), nanoliposomes, phytosomes, noisome polymer nanoparticles, nanomicelles, and carbon nanotubes, which are used in drug delivery systems, significantly increase the effectiveness, and improve the pharmacokinetics of drugs with reduced side effects [90]. Many reports revealed that several nanoformulations from a number of natural products, such as emblicanin-A and emblicanin-B, quercetin, curcumin, piperine, nigella, etc., have become a promising technology for the use of nanoformulation from natural products, as shown in Figure 4 [88,91].

### 3.5. Nanoformulation of Emblica officinalis and Its Applications

Nanomedicine has developed into a successful platform that incorporates various modalities, including therapeutics and diagnostics. It might provide individualized medical treatment to manage fatal illnesses such as cancer and diabetes. Indeed, nanocarriers act as precise delivery mechanisms for desired phytochemicals to the target site and are biocompatible, biodegradable, and less toxic. The site-specific, controlled, slow, and sustained delivery of phyto-based drugs by nanoparticles (NPs) with exceptional entrapment efficiency may enhance both their pharmacokinetics and bioavailability, while also increasing membrane permeability and preventing drug efflux through gastrointestinal mucosa [91]. Recently, there has been an increase in interest in using nanotechnology to boost phytochemical effectiveness. In the healthcare industry, the advent of nanotechnology in medical therapeutic strategies has raised hopes for the delivery of better treatments with greater efficacy and precision [92,93,94]. Nanoformulation of *E. offficinalis* is the major research area of interest due to its synergistic and improved bioavailability efficacy. The study by Omran et al. [95] focused on the synthesis of nanoemulgel by adding Carbopol 940 along with *E. officinalis* and other extracts to improve the synergistic efficacy of the extract for their antimicrobial property. Silver nanoformulation amla were studied for their antiproliferative and cytotoxic activity by Rosario et al. [96] and Abitha et al. [97]. Biosynthesis of nanocomposites using silver and graphene oxide and *E. officinalis* were characterized and studied for their antibacterial and cytotoxicity activities [98]. A recent study by Ranjani et al. [99] shows the significant cytotoxicity and antibacterial activity of amla-mediated graphene oxide and silver nanocomposites against oral pathogens. Another survey by Naik et al. [100] exhibits the anticancer and antidiabetic activity of phytofabricated silver and zinc-oxide conjugated *E. officinalis*. Considering its eco-friendly and safe aspects, the study recommends its use for pharmaceutical applications. In addition, green synthesized amla with magnesium oxide exhibited photocatalysis activity (Evans blue degradation) and antibacterial activity, thereby confirming amla’s efficacy in the removal of water contaminants.

**Figure 5:**
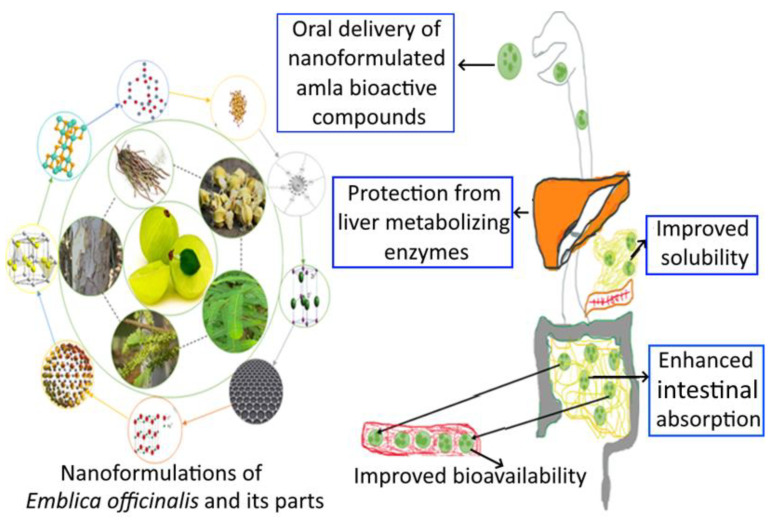
Nanoformulations of amla and its health benefits.

In contrast, phytoconstituents of *E. officinalis* such as ellagic acid, gallic acid, quercetine, and chebulagic acid were nanoformulated for the amelioration of oral bioavailability and biocompatibility properties. Harakeh et al. [101] studied the antidiabetic property of novel nanoformulated ellagic acid. Another study conducted by Hosny et al. [102] developed the sustained release of ellagic acid nanotransferosomes for its antiproliferative activity. The amla fruit’s active ingredient, gallic acid, is abundant naturally and has a variety of health benefits that makes it appealing for use in clinical settings. To increase amla’s aqueous solubility and subsequently bioactivity, gallic acid was extracted and separated from it. Using a probe sonicator and a high-pressure homogenization method, glyceryl monooleate (GMO), chitosan, and poloxamer 407 were combined to create gallic acid nanoparticles. According to the study’s findings, nanoparticles can be designed and manufactured to facilitate the extraction, manufacture, and sustained release of gallic acid, particularly in the colonic region [103]. Dendrimer nanodevices coated with gallic acid were developed to fight against chemoresistance in neuroblastoma cells [104]. Gallic acid and quercetine nanopolymers were synthesized to improve its bioavailability. Ongoing studies have concentrated on the pharmacological properties of gallic acid and its derivatives, as well as their biological effects on skin, with a particular focus on their use in (nano-)cosmetic formulations. Because the field of study is still developing, emphasis has been given to its advantages of various nanoformulations [105]. *E. officinalis* (leaves, stem, root, fruit, seeds) and its active compounds nanoformulation and delivery system are shown in Figure 5.

### 3.6. Health Care Application for Emblicanin-A and Emblicanin-B Nanoformulation

The fruit stands out from the competition due to its natural composition, which is thought to help the body fight off various illnesses and strengthen the immune system. With adequate amounts of fiber, carbohydrates, and iron, this is the best source of vitamin C. The herb *E. officinalis* is a powerful antioxidant. The fruit contains Emblicanin-A and Emblicanin-B, two hydrolyzable tannins. Ellagic acid (EA), gallic acid (GA), glucose, and EA glucose are produced during the hydrolysis of emblicanin-A and emblicanin-B [106].

The creation of a nanosized formulation has as its goal the achievement of high therapeutic efficacy with minimal toxicity. As it has better efficacy and fewer side effects, herbal medicine has long been accepted as a form of treatment by doctors for their patients. The scientific method for sustained drug delivery to the target site helps avoid repeated dosing and causes less harm to the other healthy cells or tissues. For herbal constituents, novel drug delivery systems reduce the need for repeated administration. The development of formulations with the aid of nanotechnology is one potential application. The development of herbal constituent nanoformulations relies heavily on nanocarriers [107]. The involved EA microdispersion was prepared to improve the EA’s poor water solubility and low bioavailability. The content improved nearly 30 times the water solubility and 22% (*w*/*w*) drug loading by using only water and low methoxylated pectin as a food-compatible excipient (DL). Later, non-PAMAM was used. We were successful in creating two EA nanodispersions using hydrophilic and amphiphilic (polyamidoamine) dendrimers as nanocontainers, obtaining water-solubility 300–1000 times at (60–70 nm) with 46 and 53% (*w*/*w*) DL higher than the free EA’s. Suitable for food and biomedical applications, this bioactive compound is a very effective antioxidant that is also nontoxic [108].

GA’s nanoformulation was fabricated and measured. The GA units for peripheral esterification and a delivery system that is GA-enriched (GAD) with exceptional antioxidant capacity and significant potential were successful in preventing diseases from oxidative stress (OS). GA is highly efficient against the illness that OS causes [94]. It has very few clinical applications due to inadequate gastrointestinal absorption and pharmacokinetic drawbacks, fast metabolism, and strong backs. The ready dendrimer made of polyester GA has been manufactured with an absorbable carrier to protect and deliver it. The stability in solution with a tendency to form was indicated by a ZP of 25 mV low polydispersity index and megamers. It has been on display to demonstrate GAD has four times more intrinsic antioxidant power than the GA [109].

Ellagic acid-nanosponges (EA-NS) utilized cyclodextrin and cross-linked by dimethyl carbonate is a nanoformulation that improved the solubilization efficiency of EA and controlled its release to achieve better oral absorption bioavailability. The polyphenolic compound EA, which is naturally present in many fruits, has demonstrated antioxidant, anticancer, and antimutagenic properties; however, its disadvantage is that it has a low oral bioavailability by creating a nanoformulation [109]. The use of natural product-based nanoformulations in treating various metabolic syndromes has grown in popularity among researchers. The compounds’ solubility, bioavailability, and efficacy were all improved through nanosizing. The effectiveness of a number of natural constituent nanoformulations in the treatment of numerous diseases has been observed. The molecular targets were pertinent to the way these compounds affect metabolic disorders. The natural bioactive substances emblicanin-A and emblicanin-B have high therapeutic potential and can be incorporated into systems for treating various diseases using nanotechnology [110].

The primary use of lipid-lowering medications such as statins and/or derivatives of fibric acid has been to treat elevated lipid levels and the negative effects that go along with them. It is likely that the modern medical system works to treat disease on the one hand while having negative side effects on the other [111]. The creation of lipid-lowering medications or formulations derived from natural sources has become more significant in recent years. As a result, there has been a lot of interest in using natural products with minimal side effects; *E. officinalis* is one such ingredient thought to have medicinal benefits. Recent research on nanoformulated gallic acid in in vitro models shows great lipid lowering activities [111]. Ellagic acid and 3-hydroxy-3-methylglutaryl-CoA nanoemulsion attenuates fat in in vivo models investigated by Harakeh et al. [112], and Dayar and Pechanova [113]. Because of its multimode cardio protective properties, *E. officinalis* has recently attracted new attention. It is also a powerful antioxidant that has been shown to affect how lipid metabolism is regulated.

### 3.7. Adversity and Toxicity of Nanoformulations

In recent years, the use of nanotechnology in medicine has significantly increased. When long-term or ongoing treatment is necessary for the management of metabolic diseases compliance has been regarded as a crucial factor. By providing a variety of administration methods, controlling release, enhancing biological stability, achieving target specificity, and reducing toxicity, nanoformulations have been found to increase patient compliance [114]. Accordingly, interest in creating nanoformulations to treat metabolic diseases has been dramatically increasing. Nevertheless, the majority of these studies have been limited by a lack of long-term exploratory statistics and insufficient data, particularly when it comes to the sustained resilience profiling, long-term therapeutic efficacy, and toxicological properties of the developed nanoformulations of plant-derived molecules to treat metabolic disorders. As a result, the majority of the findings are limited to the laboratory scale. Therefore, finding a solution to this problem requires considerable attention [115,116]. The toxicity evaluation of nanoscale materials and with their multiple delivery methods for active principles and nutritional supplements is of utmost priority. The use of novel functional materials could be accompanied by a number of safety concerns and require the implementation of speculative practices that consider human health and safety [117].

### 3.8. Correlation between Microbiota Bioactivity and Bioavailability of Functional Compounds: Perspectives

**Figure 6:**
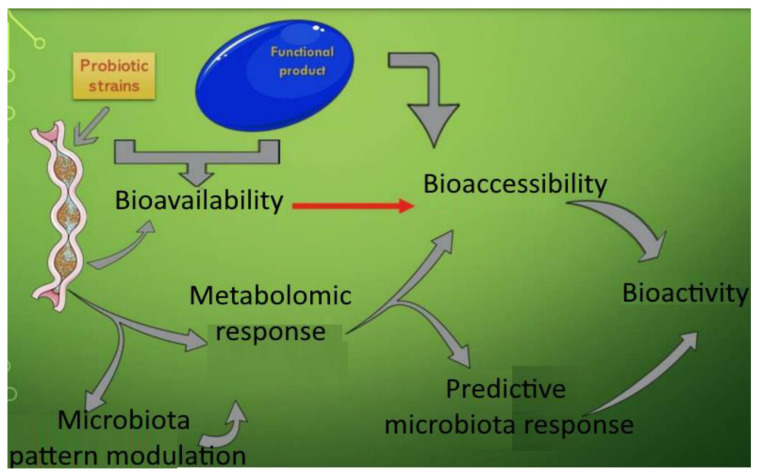
The influence of microbiota bioactivity and bioavailability of functional compounds. Figure 6 is adapted from Vamanu and Gatea [125] (Copyright © 2020 by authors), which is an open access article distributed under the terms and conditions of the Creative Commons Attribution (CC BY) license.

Cardiovascular diseases, obesity, inflammatory bowel disease, diabetes, allergies, neurological disorders, and cancer are just a few of the illnesses that the gut microbiota has been linked to in recent research [118]. The state of the gut bacteria directly affects the metabolism and bioavailability of certain nutrients, substances from food, or substances given to maintain health, such as dietary supplements based on natural substances. Some of these substances, which come from exogenous sources (such as polyphenols), may enhance the microbiota’s condition and lessen its oxidative stress [119]. Currently, there is a direct correlation between the changes or modifications of the colon’s microbial pattern and the steady rise in body weight [120], causes of which include a high caloric intake, the use of food additives and sweeteners, or the administration of other substances [121]. Genetic factors also play a role. Degenerative diseases have been linked in recent years to obesity and colon dysbiosis [122,123]. Fruits, vegetables, tea, and coffee are the main dietary sources of polyphenolic compounds, but they can also be obtained by taking various dietary supplements. The scientific community is using the information on the role of the human microbiota in maintaining the general state of health. The metabolism of various bioactive compounds resulting from food consumption and/or the administration of functional supplements determines the significance of the microbial pattern (Figure 6). Natural biocomponents (such as polyphenolic acids) are a target in the battle against chronic pathologies along with probiotics and prebiotics [124,125]. The true cause of the majority of degenerative pathologies, inflammatory progression, determines these targets, and the ongoing pressure of oxidative stress maintains them [125]. The gut–brain axis mediates interactions between the human microbiota and the central nervous system [126]. This balance is upset by the dysbiotic state, and obesity is one of the major causes of the diseases that lead to this dysbiosis at the upper level. Rebalancing can be accomplished through diet and the administration of functional products, such as pro- and prebiotics [127]. An unhealthy diet, in contrast, promotes peripheral inflammatory processes in the Western way of life and raises the risk of neuroinflammation. The rehabilitation of a dysbiotic pattern reduces the prevalence of obesity and mental illness and results in a regulation of the gut–brain axis by consuming probiotics and dietary fiber on a regular basis [128].

In order to establish homeostasis due to neurotransmitters, the balance of the gut–brain axis is extremely important. The availability of bioactive substances in the diet that control the synthesis of important metabolites, such as SCFAs, mediates the metabolic response. They are triggered by high polyphenol consumption, represent a fresh approach for future in vitro/in vivo studies, and precisely pinpoint the clinical relevance. We believe that by regulating food and consuming new active ingredients, the dynamic activity of the microbiota could be utilized to prevent the occurrence of degenerative diseases [129]. Wonder polyphenols are among the active ingredients found in *E. officinalis*. Coated or functionalized gallic acid improves bioavailability and health of intestinal microbiota. Future use of these polyphenols as functional ingredients in conjunction with pre- or probiotics will undoubtedly help to maintain the health of the gut microbiota, lowering the risk of metabolic disorders.

## 4. Conclusions

Our review focused on the amla’s pharmacological and therapeutic properties and its promising hyperlipidemic activity. The nanotechnological approach has elevated amla’s pharmacological and therapeutic activity and its phytoconstituents. Nanoformulations with Emblicanin-A, Emblicanin-B, and other constituents were created and properly tested for analgesia, anticancer, and antibacterial activity. The various *E. officinalis* nanoformulation components have shown a greater therapeutic effect after being encapsulated. Despite the fact that *E. officinalis* has a wide range of theranostic uses, it is crucial to investigate its therapeutic potential for hyperlipidemia at the cellular and molecular levels using a range of biotechnological tools and methods. The latest findings in the study of the microbiota support the role that nutrients play in controlling it. By making some natural compounds more bioavailable, it is much simpler to restore the intestinal flora than it is to use pharmaceutical alternatives, which are frequently linked to the development of diseases. We think research on how the microbiota interacts with polyphenols and other elements may have an impact on the healing process and general wellbeing of people. More clinical research is required to define the proper mechanism for lowering cholesterol levels. The development of amla and its phytoconstituent-loaded nanoformulations may lead to the development of nutraceuticals and functional food supplements that will help individuals maintain a healthier lifestyle.

## Figures and Tables

**Table 1:** Classification of hyperlipidemia ^1^.

Type	Lipoprotein Abnormality	Total Cholesterol	LDL Cholesterol	Plasma TGs	Clinical Manifestation	Population Prevalence
Familia chylomicronemia (HLP type 1)	Excess chylomicrons	Elevated	Low or normal	Elevated	Lipemia retinalis, focal neurologic symptoms, failure to thrive, recurrent epigastric pain, hepatosplenomegaly	1 in 1 million
Combined hyperlipidemia (HLP type 2)	Excess LDL and VLDL	Elevated or normal	Elevated	Normal	Physical stigmata such as xanthomas or xanthelasmas are rare	1 in 40
Dysbetalipidemia (HLP type 3)	Excess IDL elevated chylomicron remnants	Elevated	Low or normal	Elevated	Tuberous and palmar xanthomata elevations in atherogenic IDL leads to increased risk for CVD	1 in 10,000
Primary simple hyperlipidemia (HLP type 4)	Excess VLDL	Elevated or normal	Normal	Elevated	Associated with increased risk of obesity, CVD, DM2, hypertension, insulin resistance, and hyperuricemia	1 in 20
Primary mixed hyperlipidemia (HLP type 5)	Excess chylomicron and VLDL	Elevated	Normal	Elevated	Similar clinical manifestation as type 1 but develops in adulthood	1 in 600

^1^ Source: Table 1 is adapted with permission (Copyright © 2016 Taylor & Francis, Oxfordshire, England, UK) from Sharma et al. [10]. LDL, low-density lipoprotein; TGs, triglycerides; HLP, hyperlipoproteinemia; VLDL, Very-low-density lipoprotein; IDL, intermediate-density lipoprotein; CVD, cardiovascular disease; DM2, diabetes mellitus, type 2.

**Table 2:** Taxonomical classification of *E. officinalis* ^1^.

Kingdom	Plantae (Plants)
Subkingdom	*Tracheobionta* (vascular plants)
Super division	*Spermatophyta* (seed plants)
Division	Angiospermae (flowering plants)
Class	Magnoliopsida
Subclass	Rosidae
Order	Euphorbiales
Family	Euphorbiaceae
Genus	*Emblica*
Species	*officinalis* Geartn

^1^ Source: Table 2 is adapted from Hasan et al. [29], which is an open access article (Copyright © 2016 by authors) distributed under the terms and conditions of the Creative Commons Attribution (CC BY) license.

**Table 3:** Botanical description of *E. officinalis* ^1^.

Feature	Description
Habitat	Central and southern India, Pakistan, Bangladesh, Sri Lanka, Malaysia, southern China, the Mascarene Islands, Southeast Asia, and Uzbekistan.
Appearance	Medium sized deciduous tree, 8–18 m height, with thin light gray bark exfoliating in small, thin, irregular flakes.
Used parts	Dried fruits, fresh fruit, seed, leaves, root bark, and flowers.
Leaves	Simple, subsessile, closely set along the branchlets, light green, having the appearance of pinnate leaves.
Fruits	15–20 mm long and 18–25 mm wide, nearly spherical or globular, wider than long and with a small and slight conic depression on both apexes. Mesocarp is yellow, and endocarp is yellowish brown in ripened condition.
	Globose, fleshy, pale yellow with six obscure vertical furrows enclosing six trigonous seeds in three 2-seeded crustaceous cocci.
	Seedlings start bearing fruits in 7–8 years after planting, while the budded clones will start bearing fruits from the 5th year onward.
	Fresh fruits are light green, and ripe fruits turn light brown in color. The average weight of the fruit is 60–70 g.
Flowers	Greenish yellow, in axillary fascicles, unisexual, males numerous on short slender pedicels, females few, subsessile, ovary 3-celled.
Seeds	Four to six, smooth, dark brown.
Barks	Thick to 12 mm, shining grayish brown or grayish green
Flowering and fruiting	February–May and December–January
Edible part	Mesocarp and endocarp that forms the hard stone which encages the seed

^1^ Source: Table 3 is adapted from Hasan et al. [29], which is an open access article (Copyright © 2016 by authors) distributed under the terms and conditions of the Creative Commons Attribution (CC BY) license.

**Table 4:** Nutritional value of *E. officinalis* ^1^.

Chemical Components	Amount
Fruits: moisture (%)	81.20
Protein (%)	0.50
Fat (%)	0.10
Mineral matter (%)	0.10
Fiber (%)	3.40
Carbohydrate (%)	14.10
Bulk elements (mg/100 g)	Net weight
Calcium (%)	0.05
Phosphorus (%)	0.02
Iron (mg/100 g)	1.20
Vitamin C (mg/100 g)	600
Nicotinic acid (mg/100 g)	0.20

^1^ Source: Table 4 is adapted from Singh et al. [34], which is an open access article (Copyright © 2011 by the Open Science Publishers LLP, London, UK) distributed under the terms and conditions of the Creative Commons Attribution (CC BY) license.

**Table 5:** Major phyto-constituents present in *E. officinalis* and their pharmaceutical effects beyond cardioprotective and anti-hyperlipidemia ^1^.

Source	Main Active Compounds	Biological Activity
Fruit	Gallic acid	Cardioprotective, anti-hyperlipidemia
Fruit	Ellagic acid	Cardioprotective, anti-hyperlipidemia
Fruit	Emblicanin A and B	Cardioprotective, anti-hyperlipidemia
Fruit	Myricetin and Kaempferol	Cardioprotective, anti-hyperlipidemia
Fruit	Punigluconin pedunculagin	Cardioprotective, anti-hyperlipidemia
Fruit	Chebulagic acid	Cardioprotective, anti-hyperlipidemia
Fruit	Geraniin and corilagin	Cardioprotective, anti-hyperlipidemia
Fruit	Quercetin and rutin	Anti-inflammatory
Fruit	Tannins and gallic acid	Gastrointestinal activity
Fruit	Flavonoids	Antidiabetic activity
Fruit	Polyphenols	Neuroprotective
Fruit	Gallic acid	Anticancer activity

^1^ Source: Table 5 is adapted with permission (Copyright © 2016 Elsevier Ltd., Amsterdam, the Netherlands) from Variya et al. [5].

## Data Availability

Data that support the findings are available within the manuscript.

## References

[1] Ezeh K.J., Ezeudemba O. (2021). Hyperlipidemia: A Review of the Novel Methods for the Management of Lipids. Cureus.

[2] El-Tantawy W.H., Temraz A. (2019). Natural products for controlling hyperlipidemia: Review. Arch. Physiol. Biochem..

[3] Mirunalini S., Krishnaveni M. (2010). Therapeutic potential of *Phyllanthus emblica* (amla): The ayurvedic wonder. J. Basic Clin. Physiol. Pharmacol..

[4] Muzaffar K., Sofi S.A., Makroo H.A., Majid D., Dar B.N. (2022). Insight about the biochemical composition, postharvest processing, therapeutic potential of Indian gooseberry (amla), and its utilization in development of functional foods—A comprehensive review. J. Food Biochem..

[5] Variya B.C., Bakrania A.K., Patel S.S. (2016). *Emblica officinalis* (Amla): A review for its phytochemistry, ethnomedicinal uses and medicinal potentials with respect to molecular mechanisms. Pharmacol. Res..

[6] Brahm A.J., Hegele R.A. (2016). Combined hyperlipidemia: Familial but not (usually) monogenic. Curr. Opin. Lipidol..

[7] Abo-Zalam H.B., El-Denshary E.S., Abdelsalam R.M., Khalil I.A., Khattab M.M., Hamzawy M.A. (2021). Therapeutic advancement of simvastatin-loaded solid lipid nanoparticles (SV-SLNs) in treatment of hyperlipidemia and attenuating hepatotoxicity, myopathy and apoptosis: Comprehensive study. Biomed. Pharmacother..

[8] Braich A.K., Kaur G., Singh A., Dar B.N. (2022). Amla essential oil-based nano-coatings of Amla fruit: Analysis of morphological, physiochemical, enzymatic parameters, and shelf-life extension. J. Food Process. Preserv..

[9] Panahi Y., Ahmadi Y., Teymouri M., Johnston T.P., Sahebkar A. (2018). Curcumin as a potential candidate for treating hyperlipidemia: A review of cellular and metabolic mechanisms. J. Cell Physiol..

[10] Sharma K., Kumar K., Mishra N. (2016). Nanoparticulate carrier system: A novel treatment approach for hyperlipidemia. Drug Deliv..

[11] Nirosha K., Divya M., Vamsi S., Sadiq M. (2014). A review on hyperlipidemia. Int. J. Nov. Trends Pharm. Sci..

[12] Hemphill L.C. (2010). Familial hypercholesterolemia: Current treatment options and patient selection for low-density lipoprotein apheresis. J. Clin. Lipidol..

[13] Nelson R.H. (2013). Hyperlipidemia as a Risk Factor for Cardiovascular Disease. Prim. Care Clin. Off. Pract..

[14] Hur S.-H. (2014). Recent Guidelines on the Management of Blood Cholesterol: 2013 ACC/AHA Guidelines and 2014 NICE Draft Guidelines. Korean J. Med..

[15] Van Lennep J.E.R., Westerveld H.T., Erkelens D.W., van der Wall E.E. (2002). Risk factors for coronary heart disease: Implications of gender. Cardiovasc. Res..

[16] Hegele R.A., Ban M.R., Hsueh N., Kennedy B.A., Cao H., Zou G.Y., Anand S., Yusuf S., Huff M.W., Wang J. (2009). A polygenic basis for four classical Fredrickson hyperlipoproteinemia phenotypes that are characterized by hypertriglyceridemia. Hum. Mol. Genet..

[17] Zhang H.-L., Tao Y., Guo J., Hu Y.-M., Su Z.-Q. (2011). Hypolipidemic effects of chitosan nanoparticles in hyperlipidemia rats induced by high fat diet. Int. Immunopharmacol..

[18] Okerson T., Patel J., DiMario S., Burton T., Seare J., Harrison D.J. (2017). Effect of 2013 ACC/AHA Blood Cholesterol Guidelines on Statin Treatment Patterns and Low-Density Lipoprotein Cholesterol in Atherosclerotic Cardiovascular Disease Patients. J. Am. Heart Assoc..

[19] Starc T.J. (2001). Management of hyperlipidemia in children. Prog. Pediatr. Cardiol..

[20] Simons L.A. (2002). Additive effect of plant sterol-ester margarine and cerivastatin in lowering low-density lipoprotein cholesterol in primary hypercholesterolemia. Am. J. Cardiol..

[21] Harchaoui K.E.L., Visser M.E., Kastelein J.J.P., Stroes E.S., Dallinga-Thie G.M. (2009). Triglycerides and Cardiovascular Risk. Curr. Cardiol. Rev..

[22] Lee M.-R., Lim C.-J., Lee Y.-H., Park J.-G., Sonn S.K., Jung I.-H., Jeong S.-J., Lee M., Oh K.S., Yang Y. (2014). The adipokine Retnla modulates cholesterol homeostasis in hyperlipidemic mice. Nat. Commun..

[23] Castilla-Guerra L., Fernández-Moreno M.D.C., Álvarez-Suero J. (2009). Secondary stroke prevention in the elderly: New evidence in hypertension and hyperlipidemia. Eur. J. Intern. Med..

[24] Karr S. (2017). Epidemiology and management of hyperlipidemia. Am. J. Manag. Care..

[25] Merćep I., Strikić D., Slišković A.M., Reiner Ž. (2022). New Therapeutic Approaches in Treatment of Dyslipidaemia—A Narrative Review. Pharmaceuticals.

[26] Hill M.F., Bordoni B. (2022). Hyperlipidemia.

[27] Koshy S.M., Bobby Z., Jacob S.E., Ananthanarayanan P.H., Sridhar M.G., Paulose D.T. (2015). Amla prevents fructose-induced hepatic steatosis in ovariectomized rats: Role of liver FXR and LXRα. Climacteric.

[28] Petrovska B.B. (2012). Historical review of medicinal plants′ usage. Pharmacogn. Rev..

[29] Hasan M.R., Islam M.N., Islam M.R. (2016). Phytochemistry, pharmacological activities and traditional uses of *Emblica officinalis*: A review. Int. Curr. Pharm. J..

[30] Pria F.F., Islam M.S. (2019). *Phyllanthus emblica* Linn. (Amla)—A Natural Gift to Humans: An Overview. J. Dis. Med. Plants.

[31] Rai N., Tiwari L., Sharma R.K., Verma A.K. (2012). Pharmaco-botanical Profile on *Emblica officinalis* Gaertn.–A Pharmacopoeial Herbal Drug. Res. Rev. J. Bot..

[32] Gantait S., Mahanta M., Bera S., Verma S.K. (2021). Advances in biotechnology of *Emblica officinalis* Gaertn. syn. *Phyllanthus emblica* L.: A nutraceuticals-rich fruit tree with multifaceted ethnomedicinal uses. 3 Biotech.

[33] Khan K.H. (2009). Roles of *Emblica officinalis* in medicine—A review. Bot. Res. Int..

[34] Singh E., Sharma S., Pareek A., Dwivedi J., Yadav S., Sharma S. (2011). Phytochemistry, traditional uses and cancer chemopreventive activity of Amla (*Phyllanthus emblica*): The Sustainer. J. Appl. Pharma. Sci..

[35] Bhandari P., Kamdod M. (2012). *Emblica officinalis* (*Amla*): A review of potential therapeutic applications. Int. J. Green Pharm..

[36] Fitriansyah S.N., Aulifa D.L., Febriani Y., Sapitri E. (2018). Correlation of total phenolic, flavonoid and carotenoid content of *Phyllanthus emblica* extract from Bandung with DPPH scavenging activities. Pharmacog. J..

[37] Hussain S.Z., Naseer B., Qadri T., Fatima T., Bhat T.A., Hussain S.Z., Naseer B., Qadri T., Fatima T., Bhat T.A. (2021). Anola (*Emblica officinalis*): Morphology, Taxonomy, Composition and Health Benefits. Fruits Grown in Highland Regions of the Himalayas.

[38] Kc Y., Rayamajhi S., Dangal A., Shiwakoti L.D. (2020). Phytochemical, Nutritional, Antioxidant Activity and Sensorial Characteristics of Amala (*Phyllanthus emblica* L.) Chutney. Asian Food Sci. J..

[39] Bhagat M., Gupta A., Kaul V.K. (2014). Indian gooseberry (*Emblica officinalis*): Pharmacognosy review. Utilisation and Management of Medicinal Plants.

[40] Bansal V., Sharma A., Ghanshyam C., Singla M.L. (2015). Rapid HPLC Method for determination of vitamin C, phenolic acids, hydroxycinnamic acid, and flavonoids in seasonal samples of *Emblica officinalis* juice. J. Liq. Chromatogr. Relat. Technol..

[41] Zhang L.-Z., Zhao W.-H., Guo Y.-J., Tu G.-Z., Lin S., Xin L.-G. (2003). Studies on chemical constituents in fruits of Tibetan medicine *Phyllanthus emblica*. China J. Chin. Mater. Medica.

[42] Zhang Y.-J., Abe T., Tanaka T., Yang C.-R., Kouno I. (2002). Two New Acylated Flavanone Glycosides from the Leaves and Branches of *Phyllanthus emblica*. Chem. Pharm. Bull..

[43] Zhang Y.-J., Nagao T., Tanaka T., Yang C.-R., Okabe H., Kouno I. (2004). Antiproliferative Activity of the Main Constituents from *Phyllanthus emblica*. Biol. Pharm. Bull..

[44] Naik G.H., Priyadarsini K.I., Bhagirathi R.G., Mishra B., Mishra K.P., Banavalikar M.M., Mohan H. (2005). In vitro antioxidant studies and free radical reactions of triphala, an ayurvedic formulation and its constituents. Phytother. Res..

[45] Bhattacharya A., Chatterjee A., Ghosal S., Bhattacharya S.K. (1999). Antioxidant activity of active tannoid principles of *Emblicaofficinalis* (amla). Indian J. Exp. Biol..

[46] Habib-ur-Rehman, Yasin K.A., Choudhary M.A., Khaliq N., Atta-ur-Rahman, Choudhary M.I., Malik S. (2007). Studies on the chemical constituents of *Phyllanthus emblica*. Nat. Prod. Res..

[47] Tewari R., Kumar V., Sharma H.K. (2019). Physical and chemical characteristics of different cultivars of Indian gooseberry (*Emblica officinalis*). J. Food Sci. Technol..

[48] Parveen K., Khatkar B.S. (2015). Physico-chemical properties and nutritional composition of aonla (*Emblica officinalis*) varieties. Int. Food Res. J..

[49] Srinivasan P., Vijayakumar S., Kothandaraman S., Palani M. (2018). Anti-diabetic activity of quercetin extracted from *Phyllanthus emblica* L. fruit: In silico and in vivo approaches. J. Pharm. Anal..

[50] Zhang Y.-J., Tanaka T., Yang C.-R., Kouno I. (2001). New phenolic constituents from the fruit juice of *Phyllanthus emblica*. Chem. Pharm. Bull..

[51] Zhang Y.J., Tanaka T., Iwamoto Y., Yang C.R., Kouno I. (2001). Novel sesquiterpenoids from the roots of *Phyllanthus emblica*. J. Nat. Prod..

[52] Zhang Y.J., Tanaka T., Iwamoto Y., Yang C.R., Kouno I. (2000). Novel norsesquiterpenoids from the roots of *Phyllanthus emblica*. J Nat. Prod..

[53] Zhang Y.J., Tanaka T., Iwamoto Y., Yang C.-R., Kouno I. (2000). Phyllaemblic acid, a novel highly oxygenated norbisabolane from the roots of *Phyllanthus emblica*. Tetrahedron Lett..

[54] Baliga M.S., Shivashankara A.R., Thilakchand K.R., Baliga-Rao M.P., Palatty P.L., George T., Rao S., Watson R.S., Preedy V.R. (2019). Hepatoprotective effects of the Indian Gooseberry (*Emblica officinalis* Gaertn): A revisit. Dietary Interventions in Liver Disease: Foods, Nutrients, and Dietary Supplements.

[55] Qi W.-Y., Li Y., Hua L., Wang K., Gao K. (2013). Cytotoxicity and structure activity relationships of phytosterol from *Phyllanthus emblica*. Fitoterapia.

[56] Chugh C.A., Bharti D. (2014). Chemical characterization of antifungal constituents of *Emblica officinalis*. Allelopath. J..

[57] Nambiar S.S., Paramesha M., Shetty N.P. (2015). Comparative analysis of phytochemical profile, antioxidant activities and foam prevention abilities of whole fruit, pulp and seeds of *Emblica officinalis*. J. Food Sci. Technol..

[58] Goyal R., Patel S. (2012). *Emblica officinalis* Geart.: A Comprehensive Review on Phytochemistry, Pharmacology and Ethnomedicinal Uses. Res. J. Med. Plant.

[59] Priya G., Parminder N., Jaspreet S. (2012). Antimicrobial and antioxidant activity on *Emblica officinalis* seed extract. Int. J. Res. Ayur. Pharma..

[60] Usha T., Middha S.K., Goyal A.K., Lokesh P., Yardi V., Mojamdar L., Keni D.S., Babu D. (2015). Toxicological Evaluation of *Emblica officinalis* Fruit Extract and its Anti-inflammatory and Free Radical Scavenging Properties. Pharmacogn. Mag..

[61] Satish S., Mohana D.C., Raghavendra M.P., Raveesha K.A. (2007). Antifungal activity of some plant extracts against important seed borne pathogens of *Aspergillus* sp.. J. Agric. Technol..

[62] Saini R., Sharma N., Oladeji O.S., Sourirajan A., Dev K., Zengin G., El-Shazly M., Kumar V. (2022). Traditional uses, bioactive composition, pharmacology, and toxicology of *Phyllanthus emblica* fruits: A comprehensive review. J. Ethnopharmacol..

[63] Patil S.G., Deshmukh A.A., Padol A.R., Kale D.B. (2012). In vitro antibacterial activity of *Emblica officinalis* fruit extract by tube Dilution Method. Int. J. Toxicol. Appl. Pharmacol..

[64] Kamal R., Yadav S., Mathur M., Katariya P. (2012). Antiradical efficiency of 20 selected medicinal plants. Nat. Prod. Res..

[65] Liu G., Xiong S., Xiang S., Guo C.W., Ge F., Yang C.R., Zhang Y., Wang Y., Kitazato K. (2011). Antiviral activity and possible mechanisms of action of pentagalloylglucose (PGG) against influenza A virus. Arch. Virol..

[66] Usharani P., Merugu P.L., Nutalapati C. (2019). Evaluation of the effects of a standardized aqueous extract of *Phyllanthus emblica* fruits on endothelial dysfunction, oxidative stress, systemic inflammation and lipid profile in subjects with metabolic syndrome: A randomised, double blind, placebo controlled clinical study. BMC Complement. Altern. Med..

[67] Malik S., Suchal K., Bhatia J., Khan S.I., Vasisth S., Tomar A., Goyal S., Kumar R., Arya D.S., Ojha S.K. (2016). Therapeutic potential and molecular mechanisms of *Emblica officinalis* gaertn in countering nephrotoxicity in rats induced by the chemotherapeutic agent cisplatin. Front. Pharmacol..

[68] Purena R., Seth R., Bhatt R. (2018). Protective role of *Emblica officinalis* hydro-ethanolic leaf extract in cisplatin induced nephrotoxicity in Rats. Toxicol. Rep..

[69] Dhingra D., Joshi P., Gupta A., Chhillar R. (2012). Possible Involvement of Monoaminergic Neurotransmission in Antidepressant-like activity of *Emblica officinalis* Fruits in Mice. CNS Neurosci. Ther..

[70] Suja R.S., Nair A.M.C., Sujith S., Preethy J., Deepa A.K. (2009). Evaluation of immunomodulatory potential of *Emblica officinalis* fruit pulp extract in mice. Indian J. Anim. Res..

[71] Ansari A., Shahriar S.Z., Hassan M., Das S.R., Rokeya B., Haque A., Haque E., Biswas N., Sarkar T. (2014). *Emblica officinalis* improves glycemic status and oxidative stress in STZ induced type 2 diabetic model rats. Asian Pac. J. Trop. Med..

[72] Akhtar M.S., Ramzan A., Ali A., Ahmad M. (2011). Effect of Amla fruit (*Emblica officinalis* Gaertn.) on blood glucose and lipid profile of normal subjects and type 2 diabetic patients. Int. J. Food Sci. Nutr..

[73] Gopa B., Bhatt J., Hemavathi K.G. (2012). A comparative clinical study of hypolipidemic efficacy of Amla (*Emblica officinalis*) with 3- hydroxy-3-methylglutaryl-coenzyme-a reductase inhibitor simvastatin. Indian J. Pharmacol..

[74] Variya B.C., Bakrania A.K., Chen Y., Han J., Patel S.S. (2018). Suppression of abdominal fat and anti-hyperlipidemic potential of Emblica officinalis: Upregulation of PPARs and identification of active moiety. Biomed. Pharmacother..

[75] Husain I., Zameer S., Madaan T., Minhaj A., Ahmad W., Iqubaal A., Ali A., Najmi A.K. (2019). Exploring the multifaceted neuroprotective actions of *Emblica officinalis* (Amla): A review. Metab. Brain Dis..

[76] Shubhi M., Rohitash J., Radhey S., Dharmendra K.M., Kshipra M., Rajashree P., De R., Mukhopadhyay A., Srivastava A.K., Shoma P.N. (2011). Anti-Helicobacter pylori and antioxidant properties of *Emblica officinalis* pulp extract: A potential source for therapeutic use against gastric ulcer. J. Med. Plants Res..

[77] Al-Rehaily A.J., Al-Howiriny T.S., Al-Sohaibani M.O., Rafatullah S. (2002). Gastroprotective effects of ‘Amla’*Emblica officinalis* on in vivo test models in rats. Phytomedicine.

[78] Santoshkumar J., Manjunath S., Sakhare P.M. (2013). A study of anti-hyperlipedemia, hypolipedemic and anti-atherogenic activity of fruit of *Emblica officinalis* (amla) in high fat fed Albino rats. Int. J. Med. Res. Health Sci..

[79] Kapoor M.P., Suzuki K., Derek T., Ozeki M., Okubo T. (2020). Clinical evaluation of *Emblica officinalis* Gatertn (Amla) in healthy human subjects: Health benefits and safety results from a randomized, double-blind, crossover placebo-controlled study. Contemp. Clin. Trials Commun..

[80] Gul M., Liu Z.-W., Haq I.U., Rabail R., Faheem F., Walayat N., Nawaz A., Shabbir M.A., Munekata P.E.S., Lorenzo J.M. (2022). Functional and Nutraceutical Significance of Amla (*Phyllanthus emblica* L.): A Review. Antioxidants.

[81] Kim H.Y., Okubo T., Juneja L.R., Yokozawa T. (2010). The protective role of amla (*Emblica officinalis* Gaertn.) against fructose-induced metabolic syndrome in a rat model. Br. J. Nutr..

[82] Nisar M.F., He J., Ahmed A., Yang Y., Li M., Wan C. (2018). Chemical components and biological activities of the genus *Phyllanthus*: A review of the recent literature. Molecule.

[83] Kaur J., Kaur D., Singh H., Khan M.U. (2013). *Emblica officinalis*: A meritocratic drug for treating various disorders. Indo Am. J. Pharm. Res..

[84] Zahid S., Anjum K.M., Mughal M.S., Yaqub A., Yameen M. (2018). Evaluation of antioxidant and antihyperlipidemic activity of Indian gooseberry (*Emblica officinalis*) fruit in high fat-fed rabbits. JAPS J. Animal Plant Sci..

[85] Iyer U., Shah A., Venugopal S. (2021). Amla (*Emblica officinalis*) and Guava (*Psidium guajava*) supplementation: Impact of Low Carbon footprint local seasonal fruits on Lipemic Status of Morning Walkers. Eco. Environ. Cons..

[86] Sandeep B.S., Panda N., Sethy K., Nath S. (2022). Effect of dietary supplementation of amla (*Emblica officinalis*) powder and equivalent synthetic vitamin C on growth performance in black rock broiler chicken. Pharma Innov. J..

[87] Dabulici C.M., Sârbu I., Vamanu E. (2020). The Bioactive Potential of Functional Products and Bioavailability of Phenolic Compounds. Foods.

[88] Taleuzzaman M., Mahapatra D.K., Gupta D.K. (2021). Emblicanin-A and Emblicanin-B: Pharmacological and Nano-Pharmacotherapeutic Perspective for Healthcare Applications. Applied Pharmaceutical Practice and Nutraceuticals.

[89] Li T., Liang W., Xiao X., Qian Y. (2018). Nanotechnology, an alternative with promising prospects and advantages for the treatment of cardiovascular diseases. Int. J. Nanomed..

[90] Han H.S., Koo S.Y., Choi K.Y. (2022). Emerging nanoformulation strategies for phytocompounds and applications from drug delivery to phototherapy to imaging. Bioact. Mater..

[91] Moradi S.Z., Momtaz S., Bayrami Z., Farzaei M.H., Abdollahi M. (2020). Nanoformulations of Herbal Extracts in Treatment of Neurodegenerative Disorders. Front. Bioeng. Biotechnol..

[92] Mitchell M.J., Billingsley M.M., Haley R.M., Wechsler M.E., Peppas N.A., Langer R. (2021). Engineering precision nanoparticles for drug delivery. Nat. Rev. Drug Discov..

[93] Kapoor-Narula U., Lenka N. (2022). Phytochemicals and their nanoformulation in sustained drug delivery and therapy. Innovations in Fermentation and Phytopharmaceutical Technologies.

[94] Enrico C. (2019). Nanotechnology-Based Drug Delivery of Natural Compounds and Phytochemicals for the Treatment of Cancer and Other Diseases. Stud. Nat. Prod. Chem..

[95] Omran Z., Bader A., Porta A., Vandamme T., Anton N., Alehaideb Z., El-Said H., Faidah H., Essa A., Vassallo A. (2020). Evaluation of Antimicrobial Activity of Triphala Constituents and Nanoformulation. Evid.-Based Complement. Altern. Med..

[96] Rosarin S., Fathima S., Vadivel A., Samuthira N., Sankaran M. (2013). Antiproliferative effect of silver nanoparticles synthesized using amla on Hep2 cell line. Asian Pac. J. Trop Med..

[97] Abitha S.T., Rajeshkumar S., Lakshmi T., Roy A. (2019). Cytotoxic effects of silver nanoparticles synthesized using amla fruit seed. Drug Invent. Today.

[98] Soundarajan S., Sankari M., Rajeshkumar S. (2020). Antibacterial activity and cytotoxicity of amla seed mediated graphene oxide, silver nanoparticle and go-ag nanoparticle-an in vitro study. Plant Cell Biotechnol. Mol. Biol..

[99] Ranjani S., Hemalatha S. (2021). Triphala decorated multipotent green nanoparticles and its applications. Mater. Lett..

[100] Naik S., Jarnain T., David N. (2022). Phytofabrication of silver and zinc oxide nanoparticles using the fruit extract of *Phyllanthus emblica* and its potential anti-diabetic and anti-cancer activity. Part Sci. Technol..

[101] Harakeh S., Mohammed A., Al Soad J., Saad A., Saber H., Al Turki A., Najiah, Azhar E. (2020). Antidiabetic effects of novel ellagic acid nanoformulation: Insulin-secreting and anti-apoptosis effects. Saudi J. Biol. Sci..

[102] Hosny K.M., Rizg W.Y., Khallaf R.A. (2020). Preparation and Optimization of In Situ Gel Loaded with Rosuvastatin-Ellagic Acid Nanotransfersomes to Enhance the Anti-Proliferative Activity. Pharmaceutics.

[103] Patil P., Suresh K. (2021). Chitosan and glyceryl monooleate nanostructures containing gallic acid isolated from amla fruit: Targeted delivery system. Heliyon.

[104] Alfei S., Barbara M., Guendalina Z., Federica T., Cinzia D. (2020). Dendrimer nanodevices and gallic acid as novel strategies to fight chemoresistance in neuroblastoma cells. Nanomaterials.

[105] Khan B.A., Mahmood T., Menaa F., Shahzad Y., Yousaf A.M., Hussain T., Ray S.D. (2018). New perspectives on the efficacy of gallic acid in cosmetics & nanocosmeceuticals. Curr. Pharm. Des..

[106] Alfei S., Turrini F., Catena S., Zunin P., Parodi B., Zuccari G., Pittaluga A.M., Boggia R. (2019). Preparation of ellagic acid micro and nano formulations with amazingly increased water solubility by its entrapment in pectin or non-PAMAM dendrimers suitable for clinical applications. New J. Chem..

[107] Alfei S., Catena S., Turrini F. (2019). Biodegradable and biocompatible spherical dendrimer nanoparticles with a gallic acid shell and a double-acting strong antioxidant activity as potential device to fight diseases from “oxidative stress”. Drug Deliv. Transl. Res..

[108] Mady F., Ibrahim S.R.-M. (2018). Cyclodextrin-based nanosponge for improvement of solubility and oral bioavailability of Ellagic acid. Pak. J. Pharm. Sci..

[109] Rosman R., Saifullah B., Maniam S., Dorniani D., Hussein M.Z., Fakurazi S. (2018). Improved Anticancer Effect of Magnetite Nanocomposite Formulation of Gallic Acid (Fe3 O4 -PEG-GA) Against Lung, Breast and Colon Cancer Cells. Nanomaterials.

[110] Klooster S.T., Villeneuve P., Bourlieu-Lacanal C., Durand E., Schroën K., Berton-Carabin C. (2022). Alkyl chain length modulates antioxidant activity of gallic acid esters in spray-dried emulsions. Food Chem..

[111] Pathak K., Das R.J., Saikia R., Sahariah J.J., Pathak H., Sarma H., Das A. (2022). Design and fabrication of gallic acid loaded chitosan nanoformulation. Drug Deliv. Lett..

[112] Harakeh S., Qari M., Rajeh N., Ali S., El-Shitany N., Hassan S., Abd-Allah E.A., Tashkandi H., Malik M.F.A., Aljabri F.K. (2022). Ellagic acid nanoparticles attenuate oxidative stress and testicular damage in high fat Diet/Streptozotocin-Induced diabetic rats. J. King Saud Univ. Sci..

[113] Dayar E., Pechanova O. (2022). Targeted Strategy in Lipid-Lowering Therapy. Biomedicines.

[114] Dewanjee S., Chakraborty P., Mukherjee B., De Feo V. (2020). Plant-Based Antidiabetic Nanoformulations: The Emerging Paradigm for Effective Therapy. Int. J. Mol. Sci..

[115] Taghipour Y.D., Hajialyani M., Naseri R., Hesari M., Mohammadi P., Stefanucci A., Mollica A., Farzaei M.H., Abdollahi M. (2019). Nanoformulations of natural products for management of metabolic syndrome. Int. J. Nanomed..

[116] Martakov I.S., Shevchenko O.G., Torlopov M.A., Gerasimov E.Y., Sitnikov P.A. (2019). Formation of gallic acid layer on γ-AlOOH nanoparticles surface and their antioxidant and membrane-protective activity. J. Inorg. Biochem..

[117] Puttasiddaiah R., Lakshminarayana R., Somashekar N.L., Gupta V.K., Inbaraj B.S., Usmani Z., Raghavendra V.B., Sridhar K., Sharma M. (2022). Advances in Nanofabrication Technology for Nutraceuticals: New Insights and Future Trends. Bioengineering.

[118] Wang B., Yao M., Lv L., Ling Z., Li L. (2017). The Human Microbiota in Health and Disease. Engineering.

[119] Vamanu E. (2019). Polyphenolic Nutraceuticals to Combat Oxidative Stress Through Microbiota Modulation. Front. Pharmacol..

[120] Scotti E., Boué S., Sasso G.L., Zanetti F., Belcastro V., Poussin C., Sierro N., Battey J., Gimalac A., Ivanov N.V. (2017). Exploring the microbiome in health and disease. Toxicol. Res. Appl..

[121] Guirro M., Costa A., Gual-Grau A., Herrero P., Torrell H., Canela N., Arola L. (2019). Effects from diet-induced gut microbiota dysbiosis and obesity can be ameliorated by fecal microbiota transplantation: A multiomics approach. PLoS ONE.

[122] Kho Z.Y., Lal S.K. (2018). The Human Gut Microbiome—A Potential Controller of Wellness and Disease. Front. Microbiol..

[123] Mileo A.M., Nisticò P., Miccadei S. (2019). Polyphenols: Immunomodulatory and Therapeutic Implication in Colorectal Cancer. Front. Immunol..

[124] Kawabata K., Yoshioka Y., Terao J. (2019). Role of Intestinal Microbiota in the Bioavailability and Physiological Functions of Dietary Polyphenols. Molecules.

[125] Vamanu E., Gatea F. (2020). Correlations between Microbiota Bioactivity and Bioavailability of Functional Compounds: A Mini-Review. Biomedicines.

[126] Yu J.B., Zhao Z.X., Peng R., Pan L.B., Fu J., Ma S.R., Han P., Cong L., Zhang Z.W., Sun L.X. (2019). GutMicrobiota-Based Pharmacokinetics and the Antidepressant Mechanism of Paeoniflorin. Front. Pharmacol..

[127] Frolinger T., Sims S., Smith C., Wang J., Cheng H., Faith J., Ho L., Hao K., Pasinetti G.M. (2019). The gut microbiota composition affects dietary polyphenols-mediated cognitive resilience in mice by modulating the bioavailability of phenolic acids. Sci. Rep..

[128] Liu W., Luo Z., Zhou J., Sun B. (2022). Gut Microbiota and Antidiabetic Drugs: Perspectives of Personalized Treatment in Type 2 Diabetes Mellitus. Front. Cell Infect. Microbiol..

[129] Liu R., Hong J., Xu X., Feng Q., Zhang D., Gu Y., Shi J., Zhao S., Liu W., Wang X. (2017). Gut microbiome and serum metabolome alterations in obesity and after weight-loss intervention. Nat. Med..

